# Synergistic Effect of Artificial Tears Containing Epigallocatechin Gallate and Hyaluronic Acid for the Treatment of Rabbits with Dry Eye Syndrome

**DOI:** 10.1371/journal.pone.0157982

**Published:** 2016-06-23

**Authors:** Ching-Li Tseng, Ya-Jung Hung, Zhi-Yu Chen, Hsu-Wei Fang, Ko-Hua Chen

**Affiliations:** 1 Graduate Institute of Biomedical Materials and Tissue Engineering, College of Biomedical Engineering, Taipei Medical University, No. 250, Wu-Hsing Street, Taipei City 110, Taiwan; 2 Department of Chemical Engineering and Biotechnology, National Taipei University of Technology, No.1, Section 3, Chung-Hsiao E. Road, Taipei City 106, Taiwan; 3 Institute of Biomedical Engineering and Nanomedicine Research, National Health Research Institutes, Taiwan, No. 35, Keyan Rd., Zhunan Town, Miaoli County 350, Taiwan; 4 Department of Ophthalmology, Taipei Veterans General Hospital, No. 201, Sec. 2, Shipai Rd., Beitou District, Taipei City 112, Taiwan; 5 Department of Ophthalmology, School of Medicine, College of Medicine, Taipei Medical University, No. 250, Wu-Hsing Street, Taipei City 110, Taiwan; Cedars-Sinai Medical Center; UCLA School of Medicine, UNITED STATES

## Abstract

Dry eye syndrome (DES) is a common eye disease. Artificial tears (AT) are used to treat DES, but they are not effective. In this study, we assessed the anti-inflammatory effect of AT containing epigallocatechin gallate (EGCG) and hyaluronic acid (HA) on DES. Human corneal epithelial cells (HCECs) were used in the WST-8 assay to determine the safe dose of EGCG. Lipopolysaccharide-stimulated HCECs showing inflammation were treated with EGCG/HA. The expression of *IL-1ß*, *IL-6*, *IL-8*, and *TNF-α* was assessed by real-time PCR and AT physical properties such as the viscosity, osmolarity, and pH were examined. AT containing EGCG and HA were topically administered in a rabbit DES model established by treatment with 0.1% benzalkonium chloride (BAC). Tear secretion was assessed and fluorescein, H&E, and TUNEL staining were performed. Inflammatory cytokine levels in the corneas were also examined. The non-toxic optimal concentration of EGCG used for the treatment of HCECs *in vitro* was 10 μg/mL. The expression of several inflammatory genes, including *IL-1ß*, *IL-6*, *IL-8*, and *TNF-α*, was significantly inhibited in inflamed HCECs treated with 10 μg/mL EGCG and 0.1% (w/v) HA (E10/HA) compared to that in inflamed HCECs treated with either EGCG or HA alone. AT containing E10/HA mimic human tears, with similar osmolarity and viscosity and a neutral pH. Fluorescence examination of the ocular surface of mouse eyes showed that HA increased drug retention on the ocular surface. Topical treatment of DES rabbits with AT plus E10/HA increased tear secretion, reduced corneal epithelial damage, and maintained the epithelial layers and stromal structure. Moreover, the corneas of the E10/HA-treated rabbits showed fewer apoptotic cells, lower inflammation, and decreased IL-6, IL-8, and TNF-α levels. In conclusion, we showed that AT plus E10/HA had anti-inflammatory and mucoadhesive properties when used as topical eye drops and were effective for treating DES in rabbits.

## Introduction

Dry eye syndrome (DES) is a multifactorial disease of the tears and ocular surface that is associated with discomfort, visual disturbance, tear film instability, and potential damage to the ocular surface [1]. DES affects approximately 50–60 million people in the US, of which, approximately 10–15% are adults [2]. In Taiwan, the prevalence of DES is increasing with the aging of the population. Thus, DES may become an important disease of the elderly in the near future.

DES is associated with inflammation of the ocular surface and tear hyperosmolarity is an important mediator of this inflammation [1,3]. Overexpression of proinflammatory cytokines on the ocular surface has been observed in patients with DES [4,5]. Several inflammatory mediators such as interleukin (IL)-1β, IL-6, IL-17, interferon-γ, tumor necrosis factor α (TNF-α), chemokine (C-C motif) ligand 2 (CCL2), and matrix metalloproteinases have been implicated in DES-associated inflammation of the ocular surface [6,7]. Overexpression of chemokines in patients with DES induces the recruitment of leukocytes and autoreactive T cells to the ocular surface. DES also induces the production of autoimmune T cells that antagonize regulatory T cells and peripheralize to the ocular surface, resulting in epithelial damage.

Polyphenols may be involved in various processes associated with vision physiology and eye health [8,9]. Examination of the corneal damage in rabbits receiving topical phenolic acids 3 days before and 5 days after controlled UV-B exposure showed that topical application of phenolic acids significantly decreased DNA damage in the cornea and sclera tissue when compared to saline [8]. Green tea has anti-inflammatory and anti-oxidant properties [10,11]. One study showed that epigallocatechin gallate (EGCG), a principal component of green tea, inhibits inflammation associated with autoimmune disorders [11]. Some studies suggest that EGCG exerts therapeutic effects against various inflammatory diseases such as atherosclerosis, arthritis, and DES [10–12].

Ophthalmic solutions are popular because they are cost effective, have simple formulations that are easy to produce, and are well accepted by patients. However, rapid, short-term entry of these solutions into the systemic circulation is unavoidable. Therefore, the formulation must be carefully considered. In a previous study, topical ophthalmic treatment with EGCG reduced the clinical signs and inflammatory changes associated with DES by suppressing the expression of inflammatory cytokines and preventing the infiltration of CD11b^+^ cells in the murine cornea [3]. In addition, topical treatment with 0.1% EGCG significantly decreased the relative expression of IL-1β and CCL2 in rats with DES [3]. Based on these findings, in the present study, we used EGCG to treat DES. However, studies have shown that less than 5% of the dose of topically administered drugs penetrates the cornea and reaches the intraocular tissues and that a major fraction of the administered dose is absorbed systemically through the conjunctiva and nasolacrimal duct [13]. Mucus in the eye, which lubricates the corneal and conjunctival epithelial surfaces during eyelid blinking, stabilizes the preocular tear film, and prevents the entry of pathogens, is in part responsible for the limited cornea penetration. Hyaluronic acid (HA), a natural polysaccharide with superior mucoadhesive properties, has been shown to increase ocular retention time. In addition, the combination of HA and chitosan nanoparticles has been successfully used to transfer genes into the ocular tissues at a high transfection rate [14]. Therefore, we included HA in our formulation to increase drug delivery to ocular tissues.

We hypothesized that artificial tears (AT) containing EGCG and HA have a better therapeutic effect against DES than AT alone due to the anti-inflammatory effects of EGCG and the mucoadhesive properties of HA, which would inhibit proinflammatory mediators and increase the retention of the drug on the ocular surface, respectively. To test this hypothesis, we used a well-characterized rabbit model of DES.

## Materials and Methods

### Chemicals and reagents

EGCG (≥95%), lipopolysaccharide (LPS), benzalkonium chloride (BAC), and hydrocortisone were purchased from Sigma-Aldrich (St. Louis, MO, USA). Sodium hyaluronate (1%) was obtained from Maxigen (Wu-gu district, New Taipei City, Taiwan). Calcium chloride, potassium chloride, sodium chloride, and sodium phosphate dibasic were obtained from Showa Chemical Industry (Meguro-ku, Tokyo, Japan). Keratinocyte-Serum Free Medium (KSFM), bovine pituitary extract (BPE), insulin, trypsin-EDTA, penicillin/streptomycin, and phosphate-buffered saline were obtained from Gibco BRL (Gaithersburg, MD, USA). Epidermal growth factor (EGF) was obtained from PeproTech (Rocky Hill, NJ, USA). A fibronectin, collagen and albumin (FNC) Coating Mix was purchased from Athena Environmental Sciences, Inc. (Baltimore, MD, USA). The Cell Counting Kit-8 (CCK-8) was obtained from Fluka (Seelze, Germany). Tetramethylrhodamine succinyl (TAMRA-NHS) ester and TRIzol reagent were obtained from Invitrogen (Carlsbad, CA, USA). The High-Capacity cDNA Reverse Transcription Kit and TaqMan Fast Universal Master Mix (2×) were obtained from Applied Biosystems (Foster City, CA, USA). Zoletil 50 and 2% Rompun solution were obtained from Virbac Animal Health (Vauvert, Nice, France) and Bayer Korea, Ltd. (Ansan-city, Kyonggi-do, Korea), respectively. Schirmer strips (Tear Touch) were obtained from Madhu Instruments (New Delhi, India). Topical anesthesia solution (0.5% Alcaine^®^) was purchased from Alcon-Couvreur N.V. (Puurs, Belgium). Fluorescein (FL) paper strips were obtained from HAAG-STREIT AG (Koniz/Bern, Switzerland). The Apo-BrdU-IHCTM In Situ DNA Fragmentation Assay Kit was obtained from BioVision (Milpitas, CA, USA). All other chemicals were purchased from Sigma-Aldrich.

### Viability of human corneal epithelial cells treated with EGCG

A human corneal epithelial cell (HCEC) line was purchased from the American Type Culture Collection (No. CRL-11135; Manassas, VA, USA). This HCECs line has been virally transformed, and is abnormal in some parameters. Before HCEC seeding, tissue culture plastics were precoated with FNC Coating Mix. The regular culture medium contained KSFM supplemented with 50 ng/mL BPE, 5 ng/mL EGF, 5 ng/mL insulin, and 500 ng/mL hydrocortisone. Cells were subcultured and maintained at 37°C in 5% CO_2_ and 95% air. The culture medium was replaced every other day. The viability of HCECs treated with various concentrations of EGCG was determined using the CCK-8 assay kit with water-soluble tetrazolium-8 (WST-8) reagent. The cells were seeded in 96-well plates (1 × 10^4^ cells/well) and cultured overnight. Next, the HCECs were incubated with EGCG (1–1,000 μg/mL) for 1 or 3 days. The culture medium was discarded and 0.2 mL of a working solution of WST-8 was added to each well. After 4 hours of incubation, the color of a small aliquot (100 μL) of the solution was quantitatively assessed using a microspectrophotometer (Infinite M200; Tecan Trading AG, Männedorf, Switzerland) at 450 nm. The reference wavelength was set at 650 nm. The percentage of viable cells was calculated by comparison to that of control cells.

### Expression of genes encoding inflammatory cytokines in HCECs

HCECs (1 × 10^5^ cells/well) were seeded in 24-well plates containing regular growth medium and were incubated for 2 days. To induce inflammation, this medium was replaced with fresh medium containing 500 ng/mL LPS. Unstimulated HCECs were used as a control. After LPS stimulation, the medium was replaced with fresh medium containing EGCG at 1 μg/mL (E1) or 10 μg/mL (E10) with or without 0.1% (w/v) HA. After 2 hours, the cells were harvested and total RNA was extracted using TRIzol reagent according to the manufacturer’s protocol. The isolated RNA was stored at -80°C until being used for reverse transcription-polymerase chain reaction (RT-PCR). The RNA concentration was adjusted to 2 μg/μL, and first strand complementary DNA (cDNA) was synthesized using the High-Capacity cDNA Reverse Transcription Kit according to the manufacturer’s instructions. Real-time PCR was performed on a StepOne Real-Time PCR System (Applied Biosystems) using TaqMan Universal PCR Master Mix (2×) and specific primers (*IL-1β* [Hs01555413m1], *IL-6* [Hs00174131m1], *IL-8* [Hs 00174103m1], *TNF-α* [Hs00174128m1], and glyceraldehyde-3-phosphate dehydrogenase [*GAPDH*; Hs99999905m1]). Relative gene expression was quantified using the ∆∆Ct method.

### Characterization of AT containing EGCG and HA

Experiments were performed to determine the optimal concentration of EGCG for the treatment of inflamed HCECs that was both non-toxic and anti-inflammatory and a final concentration of 10 μg/mL EGCG was chosen for inclusion in the AT eye drops. Next, 0.1% (w/v) HA was added to the AT mixture (this mixture is abbreviated E10/HA). The pH and osmolarity of the AT+E10/HA were determined using a pH meter (pH 510; Eutech Instruments, Singapore) and a micro-osmometer (Model 3320; Advanced Instruments, Norwood, MA, USA). Viscosity was determined using a DV-III Digital Rheometer (Brookfield, Middleboro, MA, USA) with a cone and plate geometry to evaluate the rheological properties of the AT solution at room temperature. The basal components of a 100-mL aliquot of AT solution were 0.45 g of NaCl, 0.15 g of KCl, 0.015 g of CaCl_2_, and 0.45 g of Na_2_HPO_4_. The AT solution was freshly prepared, it was preservative free and aseptic after filtration through a 0.22 μm filter for animal study.

### Analysis of ocular retention

BALB/c mice were used to monitor the retention of the drug/fluorescent dye on the ocular surface because of the space limitation in the Xenogen *In vivo* imaging system (IVIS)-200 imaging chamber (Alameda, CA, USA). The experimental procedure was approved by the Institutional Animal Care and Use Committee (IACUC) of Taipei Medical University (IACUC approval no. LAC-101-0289). Two mice were used in this test, but repeating eye-drop dosing and examination after one day of rest were performed three times. AT containing 10 μg/mL EGCG (E10) or E10 plus 0.1% HA (E10/HA) were mixed with a fluorescent dye (100 μg/mL TAMRA). The mice were anesthetized and the fluorescent dye-containing AT eye drops were directly dropped on the ocular surface. The ocular tissues of the mice were then photographed. Subtraction of tissue auto-fluorescence using narrow band emission filters decreased the background interference and improved the detection sensitivity.

### Induction of DES in rabbits

Male New Zealand White rabbits (weight, 2.5–3.5 kg) with no signs of ocular inflammation or gross abnormalities were used. All experimental procedures were approved by the Institutional Animal Care and Use Committee (IACUC) of Taipei Medical University (IACUC approval no. LAC-100-0165). The animals were housed in standard cages in a light-controlled room at a temperature of 23°C ± 2°C, relative humidity of 60% ± 10%, and a 12-h light-dark cycle (6 AM to 6 PM). Animals were given food and water *ad libitum*. All examinations and surgical procedures were performed under general anesthesia administered via an intramuscular injection of Zoletil 50 and 2% Rompun (1:2 ratio, 1 mL/kg). The DES inducement protocol was performed as previously described [15], with slight modifications. The eyes of the rabbits were treated with eye drops containing 0.1% BAC (20 μL) 3 times daily (10 AM, 2 PM, and 6 PM) for 4 weeks [16]. Clinical observations, Schirmer’s tests, and fluorescein staining were performed before and after the BAC treatment to assess DES induction.

### Topical administration of different AT solutions to treat DES

Twenty rabbits were used for this study for two repeat animal tests. After the 4-week BAC treatment, rabbits from each test were randomly divided into 5 groups (4 eyes/2 rabbits in each group) and treated with different eye drops: (1) AT, (2) 0.1% (wt/v) HA, (3) AT containing 10 μg/mL EGCG (E10), (4) AT containing E10 and 0.1% HA (E10/HA), and (5) 0.1% BAC (negative control). Rabbits who received no treatment were used as positive controls. The eye drops were administered twice daily (10 AM and 6 PM) for 3 weeks. The rabbits were then euthanized and their corneas were carefully dissected. Before euthanasia, DES in the rabbits from each group was evaluated as described below.

#### Measurement of aqueous tear production

Aqueous tear secretion was measured using Schirmer strips [15]. Briefly, the rabbits were anesthetized to immobilize them. The test was conducted by the same person at specific time points on predetermined days in a standard environment. After topical administration of 0.5% Alcaine^®^, the lower eyelid was pulled down slightly and Schirmer strips were placed on the palpebral conjunctiva, which is near the junction of the middle and outer third of the lower eyelid. After 5 min, the wetted length of the strip was measured (in millimeters). Each eye was tested twice at a 30-min interval and the average length of the wetted paper was reported.

#### Fluorescein staining

Fluorescein staining of the corneas was performed before and after DES induction [15,17]. The therapeutic effect of the different eye drops was confirmed after the 3-week treatment. After 2 μL of 1% fluorescein sodium was dropped into the conjunctival sac, the ocular surface was examined and graded under a slit lamp microscope with a cobalt blue filter (Topcon Medical Systems Inc., Oakland, NJ, USA).

#### Histological examination of the cornea

After the animals were euthanized, each cornea was divided into two parts. One part was fixed in a 3.7% formaldehyde buffer for 24 hours. The fixed specimens were then embedded in paraffin and sectioned. The sections were stained using hematoxylin and eosin (H&E) for histological examination. Terminal deoxynucleotidyl transferase-mediated deoxyuridine triphosphate nick-end labeling (TUNEL) was performed to assess apoptosis of the corneal epithelium [3,18]. The TUNEL assay was performed using the Apo-BrdU-IHCTM *In Situ* DNA Fragmentation Assay Kit, according to the manufacturer’s instructions. The sections were examined under an optical microscope (BM-1A; SAGE vision, New Taipei City, Taiwan).

#### Quantification of inflammatory cytokines in the cornea

The other part of the cornea was used for protein extraction. The corneas were weighed and separately chopped into small pieces. The tissue was solidified by immersion in liquid nitrogen and then ground into a powder. This step was repeated three times. Tissue extraction was performed according to the reagent instructions (Tissue Protein Extraction Cocktail, ThermoFisher Scientific, Inc., Waltham, MA, USA). The tissue extract was collected and the protein content was analyzed by a Coomassie protein assay (ThermoFisher Scientific) to normalize the protein content of the samples for subsequent enzyme linked immunosorbent assay (ELISA). Total protein (15 μg in 100 μL) was loaded into 96-well plates for ELISA. Rabbit TNF-α level was determined using the DY5670 kit from R&D systems (Minneapolis, MN, USA), whereas rabbit IL-8 and IL-6 levels were determined by using kits MBS2507990 and MBS812867 (MyBioSource, San Diego, CA, USA), respectively, according to the supplier’s instructions.

### Statistical analysis

All data are expressed as mean ± standard deviation (SD) from 2–3 independent experiments. Statistical differences between groups were tested by Student’s t-test or one-way analysis of variance (ANOVA) post hoc tests and by Tukey’s test or Student’s t-test using SPSS 17.0 (SPSS, Inc., Chicago, IL, USA). A probability (*p*) value of 0.05 was considered statistically significant.

## Results

### Optimal EGCG concentration in HCECs culture

**Fig 1:**
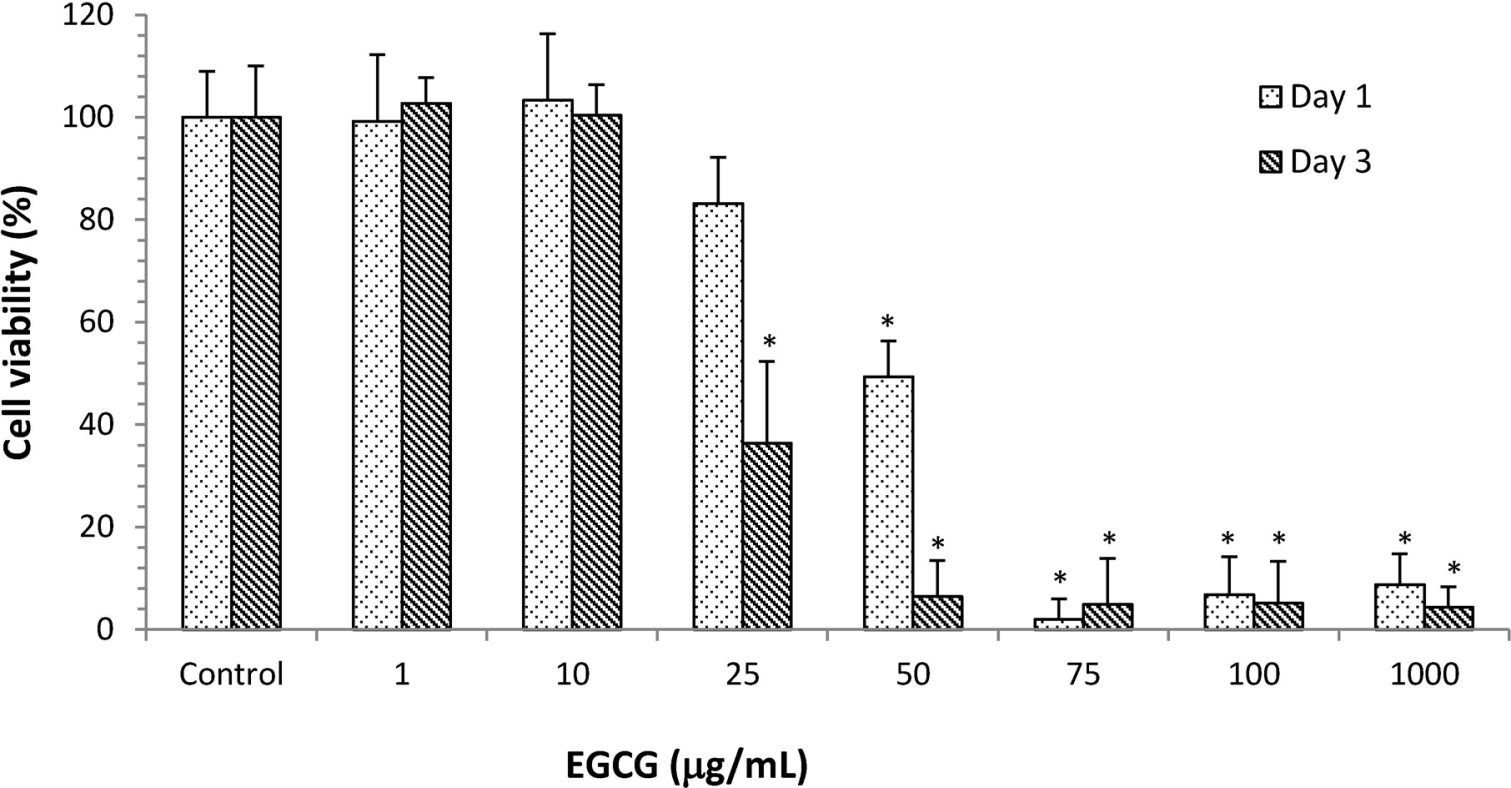
Viability of HCECs after incubation with different concentrations of EGCG. Data were analyzed using Student’s t-test and are expressed as the mean ± SD; n = 6, (*p<0.05 compared to the control group).

To evaluate the possible cytotoxic effect of EGCG, the viability of HCECs was examined in the presence of EGCG using the WST-8 assay. No toxicity was observed after 1 day of culture in the presence of EGCG at concentrations from 1 to 25 μg/mL and, at these concentrations, the percentage of viable cells was greater than 85% (Fig 1). However, the viability of HCECs incubated in the presence of ≥50 μg/mL EGCG for 1 and 3 days was significantly lower than the viability of control cells (*p< 0.05). These data indicated that EGCG concentrations less than 10 μg/mL were not toxic to HCECs.

### Anti-inflammatory effects of AT containing EGCG/HA on inflamed HCECs

**Fig 2:**
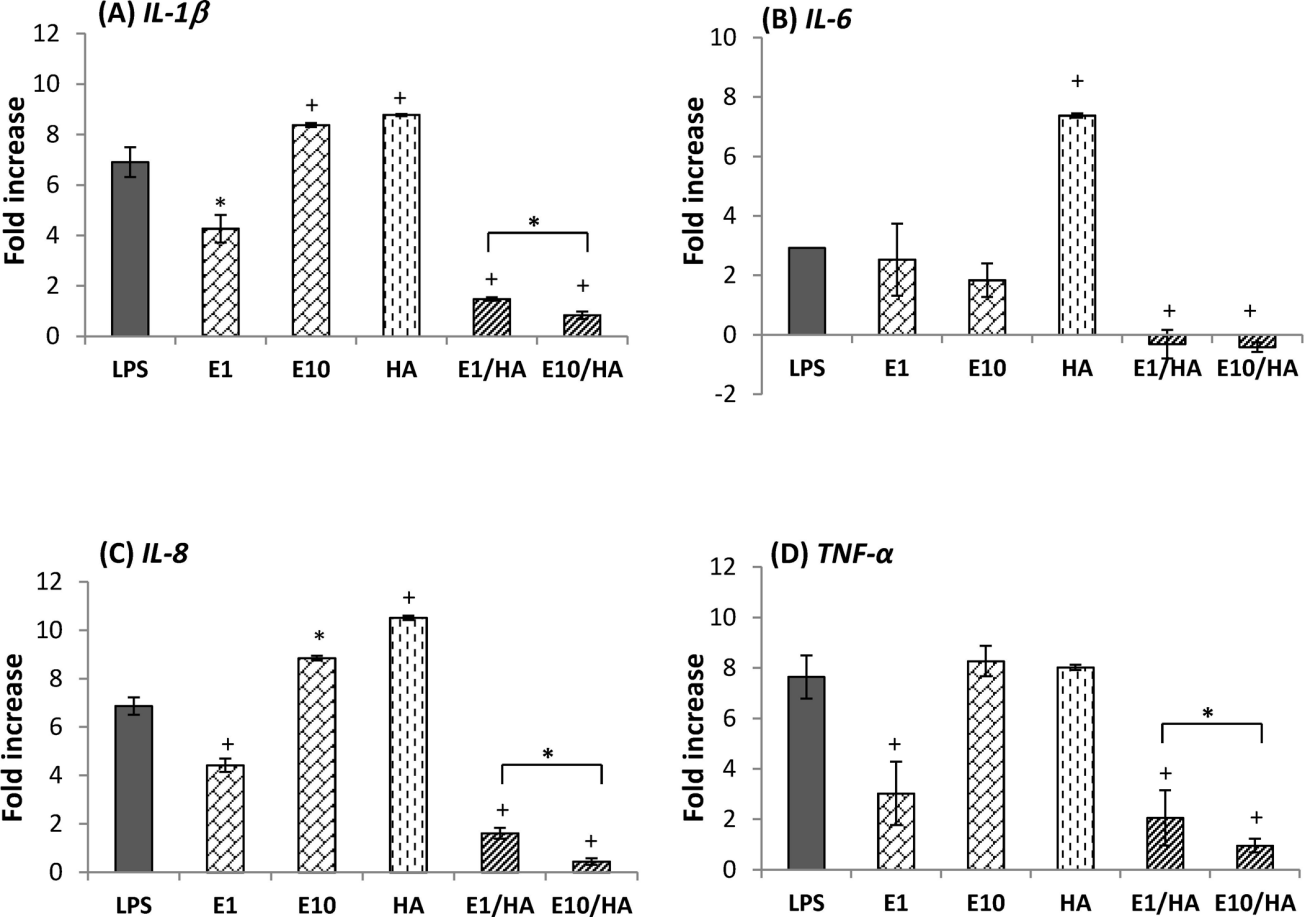
**Expression of (A) *IL-1β*, (B) *IL-6*, (C) *IL-8*, and (D) *TNF-α* in LPS-induced HCECs following various treatments.** Cells not treated with LPS were used as controls. Results are expressed as the fold increase compared with the expression in normal HCECs. All groups were compared to the LPS group for statistical analysis (n = 3, *p<0.05, +*p*<0.01). Abbreviations: LPS, lipopolysaccharide; E1, 1 μg/mL EGCG; E10, 10 μg/mL EGCG; E1/HA, E1 + 0.1% HA; E10/HA, E10 + 0.1% HA.

We examined the anti-inflammatory effect of EGCG on LPS-stimulated HCECs. Fig 2 shows the expression levels of *IL-1β*, *IL-6*, *IL-8*, and *TNF-α* in inflamed HCECs incubated with LPS, E1, E10, HA, E1/HA, and E10/HA. Compared to normal HCECs (control group, baseline levels in Fig 2), the expression of *IL-1β*, *IL-6*, *IL-8*, and *TNF-α* was increased in LPS-treated HCECs. The levels of *IL-1β*, *IL-8*, and *TNF-α* were significantly downregulated in cells treated with E1 (1 μg/mL EGCG), but were slightly upregulated in cells treated with E10 (10 μg/mL EGCG). The expression of these proinflammatory cytokines was not significantly downregulated in HA-treated cells. For example, the expression of *TNF-α* was almost the same in HA- and LPS-treated cells (8.03 ± 0.10 and 7.64 ± 0.86 fold increases compared to the control). The expression of *IL-1β* was significantly lower in cells treated with E1/HA and E10/HA than in cells treated with LPS (6.91 ± 0.56-fold). *IL-1β* expression was only 1.48 ± 0.07- and 0.83 ± 0.14-fold that of control cells, respectively. Similar trends were observed for the expression of *IL-6*, *IL-8*, and *TNF-α* in cells following treatment with E1/HA and E10/HA. Furthermore, the inhibitory effect of E10/HA on *IL-1β*, *IL-8* and *TNF-α* expression in inflamed cells was stronger than that of E1/HA (Fig 2). According to these results, the optimal concentrations of EGCG and HA for the AT solution were 10 μg/mL and 0.1%, respectively.

### Characterization of AT containing EGCG and HA

The normal pH of human tears varies between 6.5 and 7.6 [19] and the osmolarity ranges from 260–340 mOsm/kg [20]. Human tears have non-Newtonian viscosity in the range of 1–10 mPa s, whereas the viscosity of water at 20°C is 1 mPa s [21]. The pH of AT plus E10/HA was 7.6 ± 0.08 and its osmolarity was 258.0 ± 1.8 mOsm/kg (Table 1). Although these values were slightly outside the ranges for normal human tears, they were acceptable. The viscosity of the AT was in the range of 2.5–3.0 mPa s, but that of AT plus E10/HA increased to about 7 mPa s due to the addition of HA, which made it more similar to human tears.

**Table 1:** Characteristics of human tears and artificial tear fluids.

	pH	Osmotic pressure (mOsm/kg)	Viscosity (mPa s)
Normal human tears	6.5–7.6^19^	260–340^20^	1–10^21^
Artificial tear fluid	8.1 ± 0.03	245.7 ± 4.2	2.5–3.0
Artificial tear fluid with EGCG and HA*	7.6 ± 0.08	258.0 ± 1.8	6.5–7.5

*EGCG: 10 μg/mL, HA: 0.1%

### Effect of HA on the retention time of AT on the ocular surface

**Fig 3:**
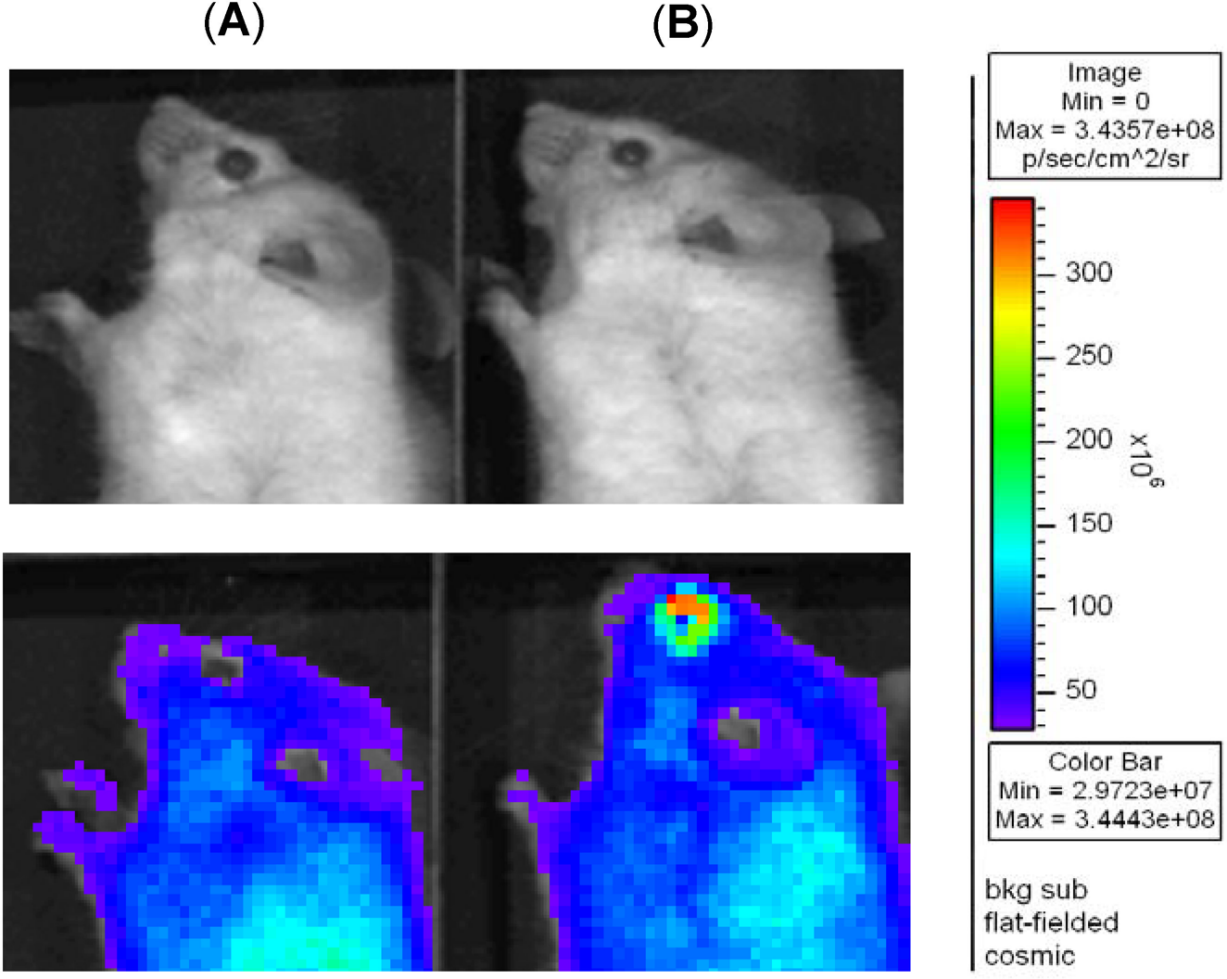
**Color photographs showing the ocular retention of the dye/drug in mice treated with (A) AT plus E10 and (B) AT plus E10/HA.** A fluorescent dye (TAMRA, 100 μg/mL) was added to both solutions for fluorescence staining. E10: 10 μg/mL; EGCG, HA: 0.1% HA.

To determine the effect of adding HA on the retention time of AT, a fluorescent dye (TAMRA) was added to AT containing E10 or E10/HA and the distribution of the eye drops in the anterior part of the eye was observed. Changes on the ocular surface of mouse eyes were examined by using an IVIS imaging system. Photographs of the eyes are shown in Fig 3. After a 15-min exposure to these AT, mice treated with AT plus E10/HA showed higher fluorescence intensity than mice treated with AT plus E10. This indicated that the addition of HA increased the retention of the dye/drug in the eyes by promoting mucoadhesion on the ocular surface.

### Therapeutic efficacy of AT solutions in the rabbit model of DES

**Fig 4:**
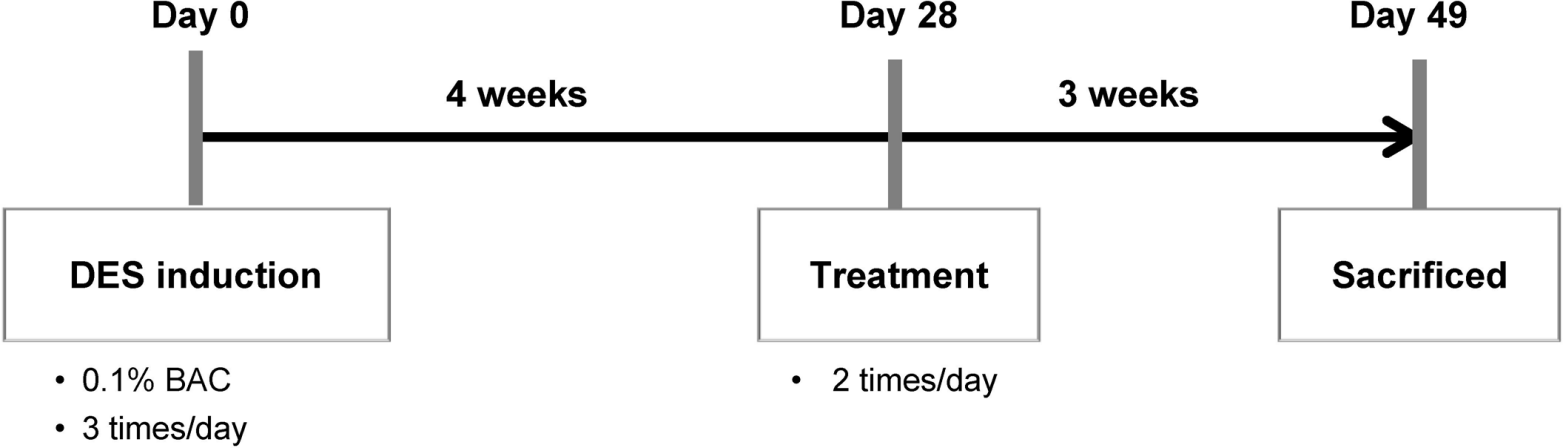
Timeline from DES induction to treatment in the rabbit model of DES.

The timeline from DES induction to treatment was as follows: First, rabbits were treated with 0.1% BAC 3 times daily for 4 weeks to establish DES. Next, rabbits with established DES were treated with different eye drops twice daily for 3 weeks (Fig 4).

#### Changes in tear production

**Fig 5:**
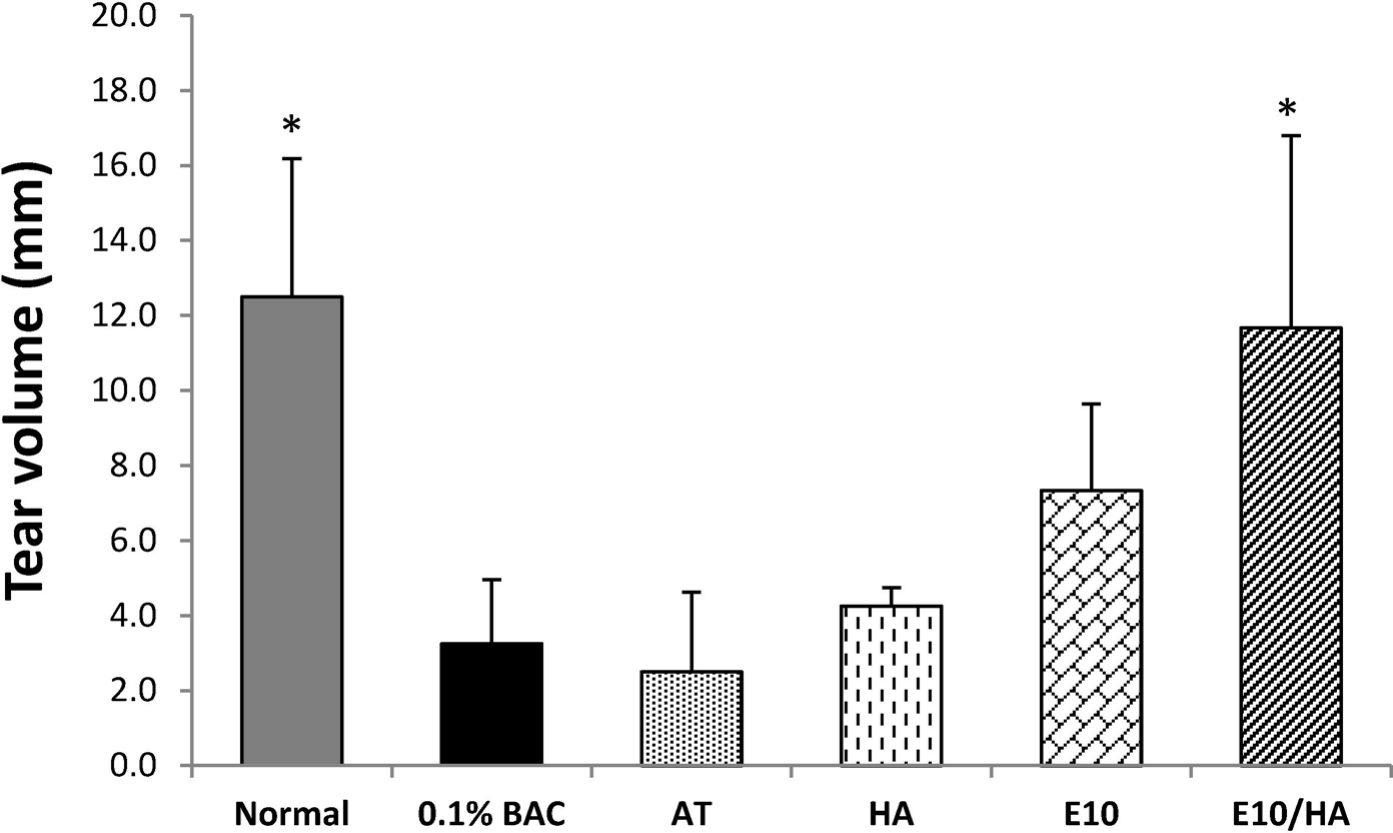
Results of Schirmer’s tests in all groups. Statistically significant differences were observed between the normal and E10/HA-treated groups after the 3-week treatment (compared with the 0.1% BAC group). Data are expressed as the mean ± SD (n = 4). *p<0.05. Abbreviations: AT, artificial tears; HA, 0.1% HA; E10, AT with 10 μg/mL EGCG; E10/HA, AT with E10 and 0.1% HA.

Tear production was examined after the 3-week eye drop treatment period. Compared with normal (non-DES) eyes, tear secretion decreased rapidly in the eyes of rabbits treated with 0.1% BAC (which mimics DES) and AT. Compared with the 0.1% BAC group, a statistically significant increase was observed in the group treated with AT plus E10/HA. However, compared with the 0.1% BAC group, no significant difference was observed in the groups treated with HA or E10 alone (Fig 5).

#### Recovery of the damaged epithelium in the cornea

**Fig 6:**
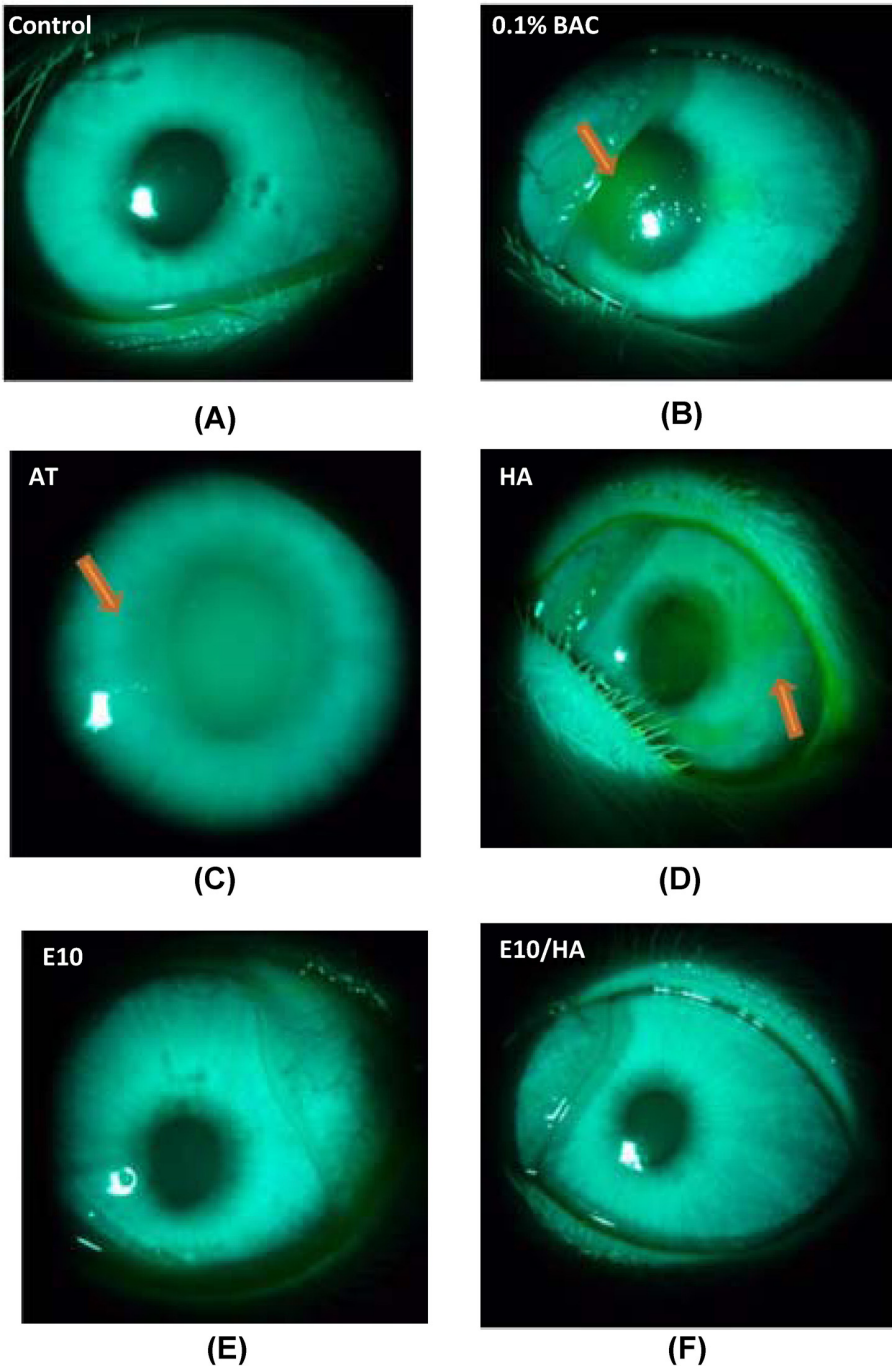
Slit lamp photographs of the rabbit eyes in each group after fluorescence staining. (A) Non-treated control eye with no fluorescent pigment, (B) eye treated with 0.1% BAC (DES group), (C) eye treated with AT, (D) eye treated with 0.1% HA, (E) eye treated with E10, and (F) eye treated with E10/HA. Damaged epithelial cells in the eyes were observed as green patches (arrows).

After the 3-week treatment period, the ocular surface of all experimental eyes was stained with fluorescein and examined under a slit lamp microscope. The eyes of normal animals were not stained with the dye (Fig 6A). In contrast, a dark green staining was observed in the center of the corneas of rabbits treated with 0.1% BAC (Fig 6B). Opaque corneas were observed in the eyes of rabbits treated with AT (Fig 6C) and a light green staining was observed in the center of the cornea of rabbits treated with HA (Fig 6D). No staining was observed in the corneas of rabbits treated with E10 and E10/HA (Fig 6E and 6F).

**Fig 7:**
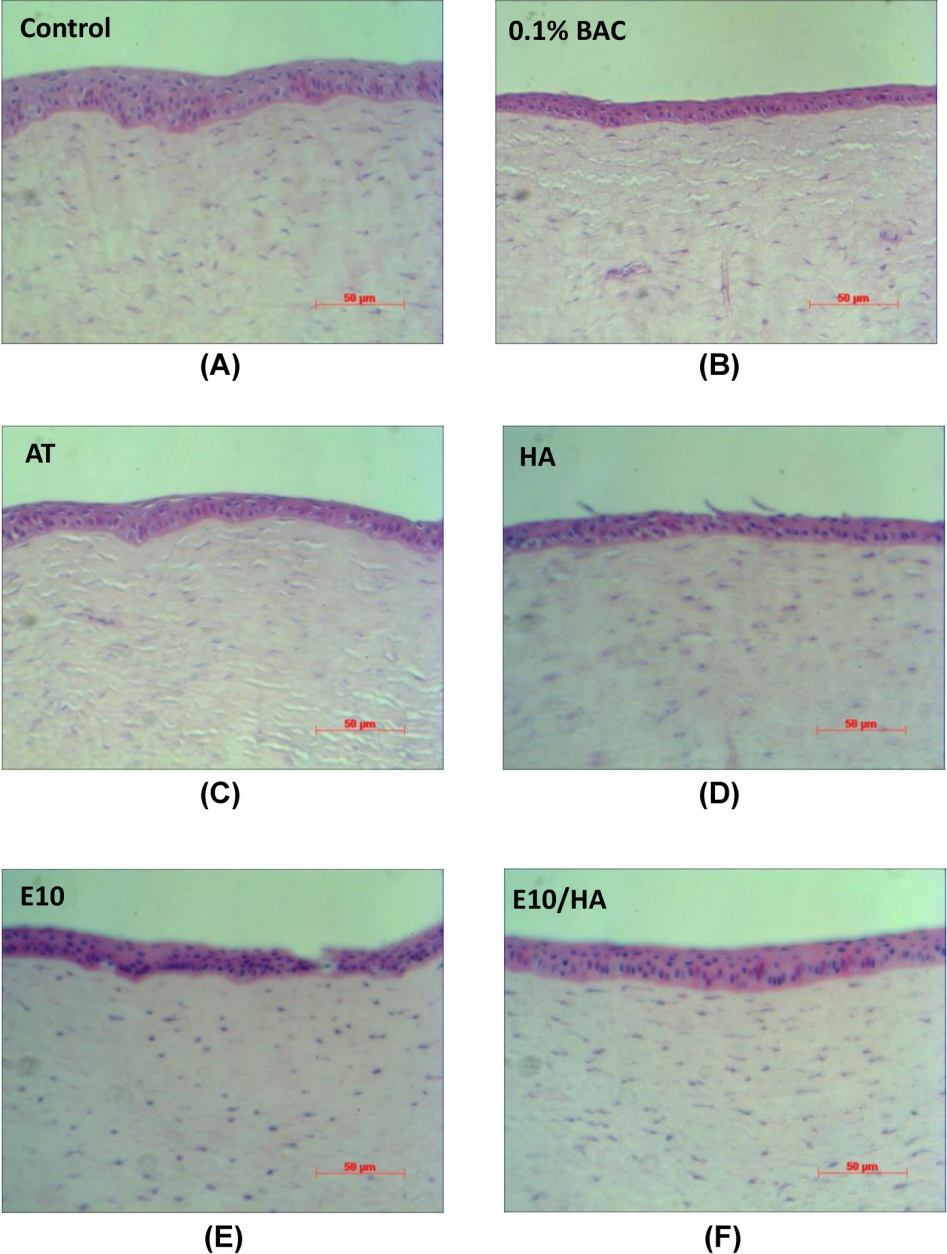
Representative pictures of H&E staining of corneal sections from the eyes of rabbits in each of the 6 groups. (A) Corneal sections from controls showing 3–5 epithelial layers. Corneal sections from (B) 0.1% BAC- and (C) AT-treated eyes showing the destruction of the layered structure and sponge-like stroma. Corneal sections from (D) 0.1% HA-, (E) E10-, and (F) E10/HA-treated eyes showing a structure similar to that in the controls. Scale bar = 50 μm

Examination under a light microscope showed that the corneas of control rabbits had 3–5 layers of epithelial cells and dense collagen fibers in the stroma (Fig 7A). Rabbits in the BAC group had a thinner corneal epithelium with only 2–3 layers and loose collagen fibers in the stroma (Fig 7B). Rabbits in the AT and HA groups had thinner corneal epithelia and loose stroma (Fig 7C and 7D). However, rabbits in the E10 and E10/HA groups presented normal corneal epithelia with a normal number of layers and thickness (Fig 7E and 7F).

**Fig 8:**
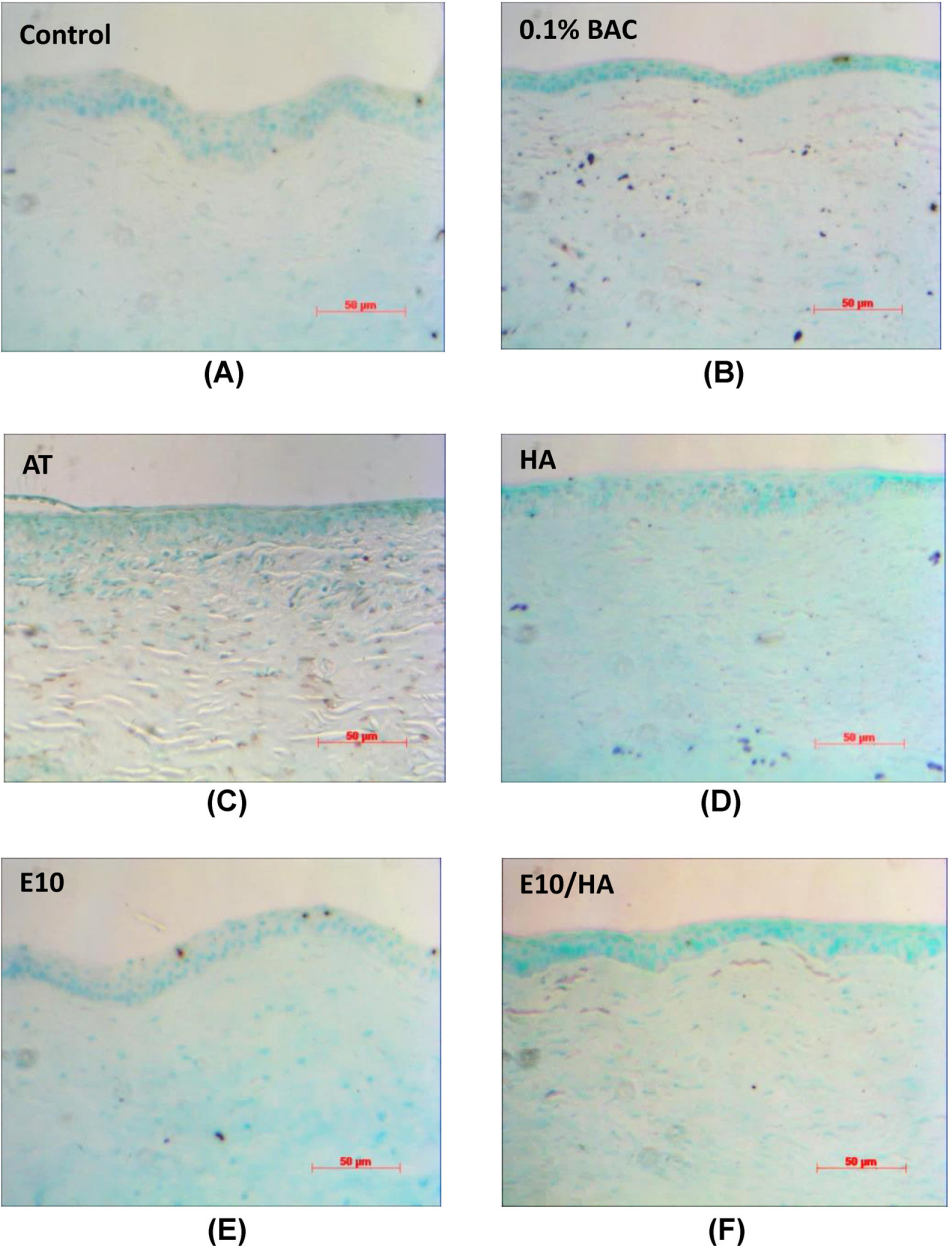
TUNEL staining of corneal sections from the eyes of rabbits in the 6 different groups. (A) Corneal sections from the eyes of controls. Corneal sections from (B) 0.1% BAC- and (C) AT-treated eyes showing positive staining (brown), indicating serious damage to the epithelial layer. Corneal sections from (D) HA-, (E) E10-, and (F) E10/HA-treated eyes showing less apoptotic cells in the apical layers, which is similar to that observed in the corneal sections from the eyes of controls. Scale bar = 50 μm.

TUNEL is a common method for detecting DNA fragmentation that results from apoptotic signaling cascades. In the TUNEL assay, cells that have suffered severe DNA damage are stained dark brown. The software “Image J” (National Institute of Health, Bethesda, MD, USA) was used to quantify the results of TUNNEL staining by setting image pixel ≥10 representing apoptotic cells with brown spots in Fig 8. Control eyes (Fig 8A) showed nearly no TUNEL-positive cells (only 6 spots were counted). In contrast, eyes from rabbits treated with 0.1% BAC showed more apoptotic cells (n = 48) in the corneal basal epithelium and stroma (Fig 8B), whereas those of rabbits treated with AT showed few apoptotic cells in the superficial corneal epithelium (about 37 apoptotic cells counted in Fig 8C). HA treatment alone did not decrease the level of apoptosis in the corneal epithelium compared with that observed in DES eyes and many apoptotic cells (n = 34) were observed in the corneal stroma (Fig 8D). In contrast, 8 apoptotic cells were counted in the eyes from rabbits treated with E10 and only 3 apoptotic cells were detected in the eyes from rabbits treated with E10/HA, which presented fewer TUNEL-positive cells (Fig 8E and 8F) than that observed in the other three groups.

#### Reduced inflammatory cytokines

**Fig 9:**
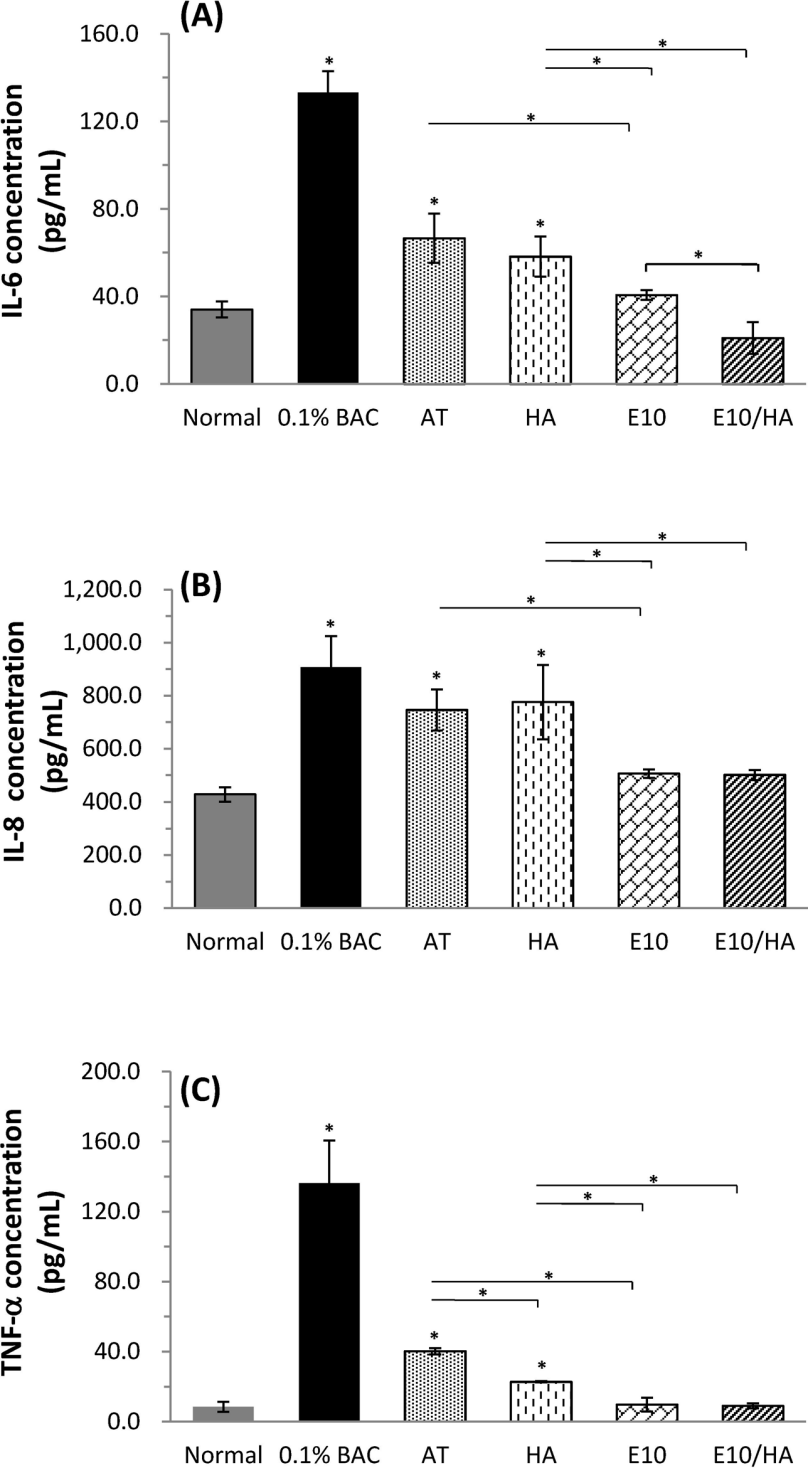
**Quantification of inflammatory cytokines in cornea extracts by ELISA**: (A) IL-6, (B) IL-8, and (C) TNF-α levels after treatment with different solutions. Data are expressed as the mean ± SD; n = 4, (*p<0.05 compared to control).

The inflamed condition of the tested cornea was assessed by measuring the expression of inflammatory cytokines in response to various treatments using ELISA as shown in Fig 9. Higher levels of IL-6, IL-8, and TNF-α were observed in corneas treated with 0.1% BAC (which mimics DES inflamed condition) compared with those in normal corneas (*p<0.05). Although IL-6 and TNF-α concentrations in the AT or AT plus HA treated groups were lower than those in the DES groups, these proinflammatory cytokine levels were still significantly higher than those observed in the normal group (*p<0.05). The concentrations of IL-6, IL-8, and TNF-α in inflamed corneas treated with E10 or E10/HA were greatly decreased compared to those in the 0.1% BAC-treated group and they did not differ significantly from the concentrations measured in the normal corneas. The levels of inflammatory cytokines in the E10 or E10/HA treated groups were significantly lower than those measured in the AT or AT plus HA treated groups (^*^p<0.05). In fact, IL-6 concentration in the E10/HA group (Fig 9A) was significant lower than that in the E10-treated group (*p<0.05). There was no significant difference in the levels of these cytokines between E10/HA-treated and normal corneas. According to these results, the AT solution with 10 μg/mL EGCG and 0.1% HA (E10/HA) could effectively inhibit inflammation in the eyes of DES rabbits.

## Discussion

Inflammation and tear hyperosmolarity have recently been added to the description of DES [22]. Therefore, the efficacy of various anti-inflammatory agents for treating DES has been examined [3,23,24]. In this study, we confirmed the synergistic effect of EGCG (anti-inflammatory agent) and HA combination for the effective treatment of rabbits with DES.

We found that EGCG at approximately 10 μg/mL (about 22 μM) was non-toxic to HCECs (Fig 1). This concentration was similar to that determined by Cavet *et al*., who showed that EGCG in the range of 0.3–30 μM was non-toxic to HCECs and that the expression of cytokines, such as IL-6, IL-8, and monocyte chemotactic protein-1, was downregulated in inflamed HCECs treated with EGCG [10]. In the present study, inflamed cells (induced by LPS stimulation) were treated with EGCG or EGCG plus HA. The combination treatment of HA and EGCG (E10/HA) significantly inhibited the expression of *IL-1β*, *IL-6*, *IL-8*, and *TNF-α* in inflamed HCECs (Fig 2). None of the previous studies examined the effectiveness of the combination of an anti-inflammatory agent and HA for treating inflamed HCECs. Thus, the present study is the first to examine the effect of EGCG plus HA on inflamed HCECs.

The osmolarity of EGCG-containing AT with or without HA was in a suitable range (255–280 mOsm/kg) and may not further harm the inflamed cornea (Table 1). The normal pH of human tears is 6.5–7.6 [19]. AT containing 10 μg/mL EGCG and 0.1% (w/v) HA (E10/HA) had a neutral pH (pH 7.6) and increased viscosity. AT plus E10/HA was tolerable and no side effect such as blurred vision, ocular redness, ocular burning, or ocular itching was reported during the treatment period (data not shown), confirming its suitability as a tear substitute for treating DES.

In the eyes, HA is part of the vitreous and aqueous humor and is a natural component of tears [25,26]. It has a high capacity to retain water and resists desiccation, which improves the wettability of the ocular surface [27]. In addition, the viscoelastic properties of mucoadhesive HA promote lubrication of the ocular surface and reduce friction during blinking and ocular movements [28]. Unlike chitosan, it does not affect the tight junctions of the corneal epithelium [14,29]. In this study, to monitor the effectiveness of HA for drug/dye retention on the ocular surface, live mice were used instead of rabbits due to limited space inside the imaging chamber. After 15 min of treatment, stronger fluorescence was observed on the ocular surface of mice treated with E10/HA compared to that on the ocular surface of mice treated with E10 alone (Fig 3). HA is involved in the regeneration of corneal and conjunctival epithelial cells by interacting with the CD44 receptor. The CD44 receptor is overexpressed in patients with DES [30,31]. Therefore, higher amount of dye/drug retention on the ocular surface with HA addition is based on the mucoadhesion and the targeting of CD44 by HA.

Mitsui *et al*. suggested that HA at a concentration of 4 mg/mL (0.4% w/v) can inhibit the expression of proinflammatory cytokines in inflammatory subacromial-synovium fibroblasts [32]. HA at low concentrations (0.1% w/v) did not effectively inhibit the inflammatory condition in HCECs (Fig 2). In Fig 9, IL-6 and TNF-α were reduced in the 0.1% HA treated group compared to the DES group, but no effect was observed on IL-8 levels. AT plus E10/HA presented *in vitro* (Fig 2) and *in vivo* (Fig 9) anti-inflammatory properties due to the presence of both EGCG and HA. The EGCG concentration in E10/HA was 10 μg/mL (0.001%), which was much lower than that used in a previous study (0.1% EGCG) on a rat model of DES [3]. Additionally, the HA concentration in E10/HA was 0.1%, which was also lower than that used in other reports as an anti-inflammatory treatment, but still presented water retention capacity on the ocular surface [32,33]. The inflammatory condition in a DES test rabbit was also alleviated following E10/HA treatment (Fig 9) due to the coordinated actions of EGCG and HA, which were effective even at low concentrations.

Several animal models have been developed to mimic different pathophysiologic mechanisms in the development of dry eye such as controlled environment at low humility (18.5% ± 5.1%) in mice [34], invasive method, including lacrimal gland removal and bulbar conjunctiva alteration using trichloroacetic acid, in rabbits [17], and topical delivery of BAC to induced DES in mice and rabbits [15,35,36]. The rabbit is the model animal for ophthalmic conditions because the exposed ocular surface is larger than that of the mouse. Therefore, standard dry eye clinical tests such as tear production and fluorescein staining of the ocular surface can be easily performed in rabbits [37].

BAC, a very common preservative used in ophthalmic agents, is a quaternary ammonium compound that has been shown to hasten the drying of tear film [38], worsen preexisting dry eye, and affect both the cornea and the conjunctiva [39]. Epidemiologic data also show that symptoms and signs of dry eye are more prevalent when BAC-preserved drops are used [40]. Besides, topical delivery of BAC to induce a dry eye condition in mice, cats, rabbits, and dogs are reported [15,35,41,42]. Although, the BAC induced DES model does not represent the whole spectrum of dry eye syndrome, the phenomena caused by BAC treatment, including the instability of the tear film, inflammation of the cornea, and damage to the tear balance condition (Figs 5, 6 and 9), are some common features of DES.

BAC destroys the tight junctions of superficial cells in the corneal epithelium [43] and this degradation of tight junctions damages the normal function of the corneal epithelium [36,44]. In the present study, fluorescein staining showed severe damage in the corneal epithelium following BAC treatment (Fig 6B). In contrast, E10/HA-treated eyes with DES did not show dye uptake (Fig 6F), indicating no damage to the corneal epithelium. BAC-induced DES leads in pathological changes in the corneal epithelium, including apoptosis and squamous metaplasia, which are closely associated with inflammation [24,36]. BAC stimulates the overexpression of inflammatory cytokines such as ICAM-1, IL-6, IL-8, and IL-10 in epithelial cells [10,36,45], which may promote apoptosis of both epithelial and goblet cells [36]. In the present study, the TUNEL assay indicated more apoptotic cells in the corneas of 0.1% BAC-treated rabbits (Fig 8B). This loss of epithelial and goblet cells may reduce mucin expression. In turn, reduced mucin production accelerates the disruption of the tear film, thus aggravating the damage to the ocular surface and stimulating an inflammatory cascade in the epithelial cells of the ocular surface [36]. In the BAC treated group, the epithelium cell layers detached and became only 2–3 layers (Fig 7B). More apoptotic cells were observed in the stroma than in the apical epithelium of 0.1% BAC-treated eyes (Fig 8B). A similar phenomenon was also revealed in a previous study indicating that corneal epithelial surface cells were detached in some areas examined by scanning electron microscopy [36]. Because BAC is a small molecule, it easily and deeply penetrates into the stromal layer causing keratocyte death (Fig 8B). Meanwhile, toxic BAC could damage the stromal keratocytes, inducing toxic medicamentosa. In this study, some rabbits presented with both dry eye and medicamentosa. The data in cultured cells should be corroborated on primary corneal cells. At the same time, the encouraging results in rabbits suggest that primary cells would behave similarly to the transformed ones.

In summary, this study showed that EGCG at 10 μg/mL was safe for treating HCECs. The expression of the inflammatory genes, *IL-1β*, *IL-6*, *IL-8*, and *TNF-α*, in inflamed HCECs was significantly downregulated when the cells were treated with a combination of 10 μg/mL EGCG and 0.1% HA (E10/HA). Characterization of AT containing E10/HA showed that they mimicked human tears, with a similar pH, osmolarity, and viscosity. Enhanced retention of EGCG/fluorescent dye on the ocular surface following the addition of HA increased the retention and bioavailability of EGCG on the ocular surface. Topical administration of AT containing E10/HA in rabbits with DES increased tear secretion, reduced damage to the corneal epithelium, maintained the normal microstructure of the cornea, and relieved the inflammation (as evidenced by decreased IL-6, IL-8, and TNF-α levels) in the corneas. Additionally, fewer apoptotic cells were observed in the corneas of E10/HA-treated dry eyes. Further clinical investigations are needed to assess the efficacy and safety of these eye drops for treating DES in humans.
